# Supplementary material for: Plant Raf-like kinases regulate the mRNA population upstream of ABA-unresponsive SnRK2 kinases under drought stress
Source: Nat Commun. 2020 Mar 13;11:1373. doi: 10.1038/s41467-020-15239-3 (PMC7069986; doi:10.1038/s41467-020-15239-3)
Supplement: Supplementary file 1 — Supplementary Information [file 41467_2020_15239_MOESM1_ESM.pdf]

Plant Raf-like kinases regulate the mRNA population upstream of ABA-unresponsive SnRK2 kinases under drought stress

Soma et al.

Supplementary Information

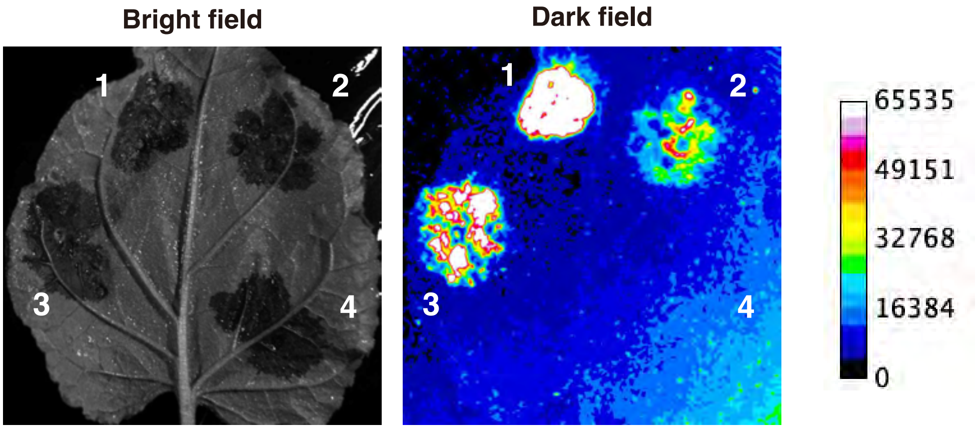

1. RAF18-nLUC + SRK2G-cLUC
2. RAF20-nLUC + SRK2G-cLUC
3. RAF24-nLUC + SRK2G-cLUC
4 nLUC + SRK2G-cLUC

Supplementary Figure 1. Split luciferase assay of the interaction between the three Raf-like kinases and SRK2G.

Split luciferase assay of the interaction between the three Raf-like kinases and SRK2G in infiltrated Nicotiana benthamiana leaves displayed by bright-field (left) and dark-field (right).

Supplementary Figure 2

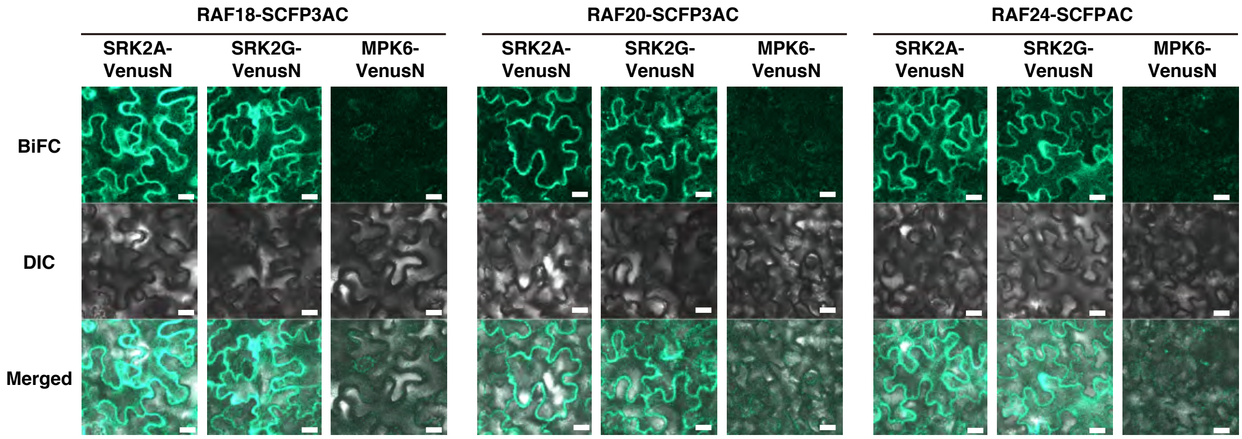

Supplementary Figure 2. BiFC analysis of the three Raf-like kinases and SRK2A or SRK2G.

BiFC analysis of the physical interactions between the three Raf-like kinases and SnRK2s in *N. benthamiana*. RAF18-SCFP3AC, RAF20-SCFP3AC or RAF24-SCFP3AC3 and SRK2A-VenusN, SRK2G-VenusN or MPK6-VenusN were transiently expressed in *N. benthamiana* leaves. Scale bars, 20 μm.

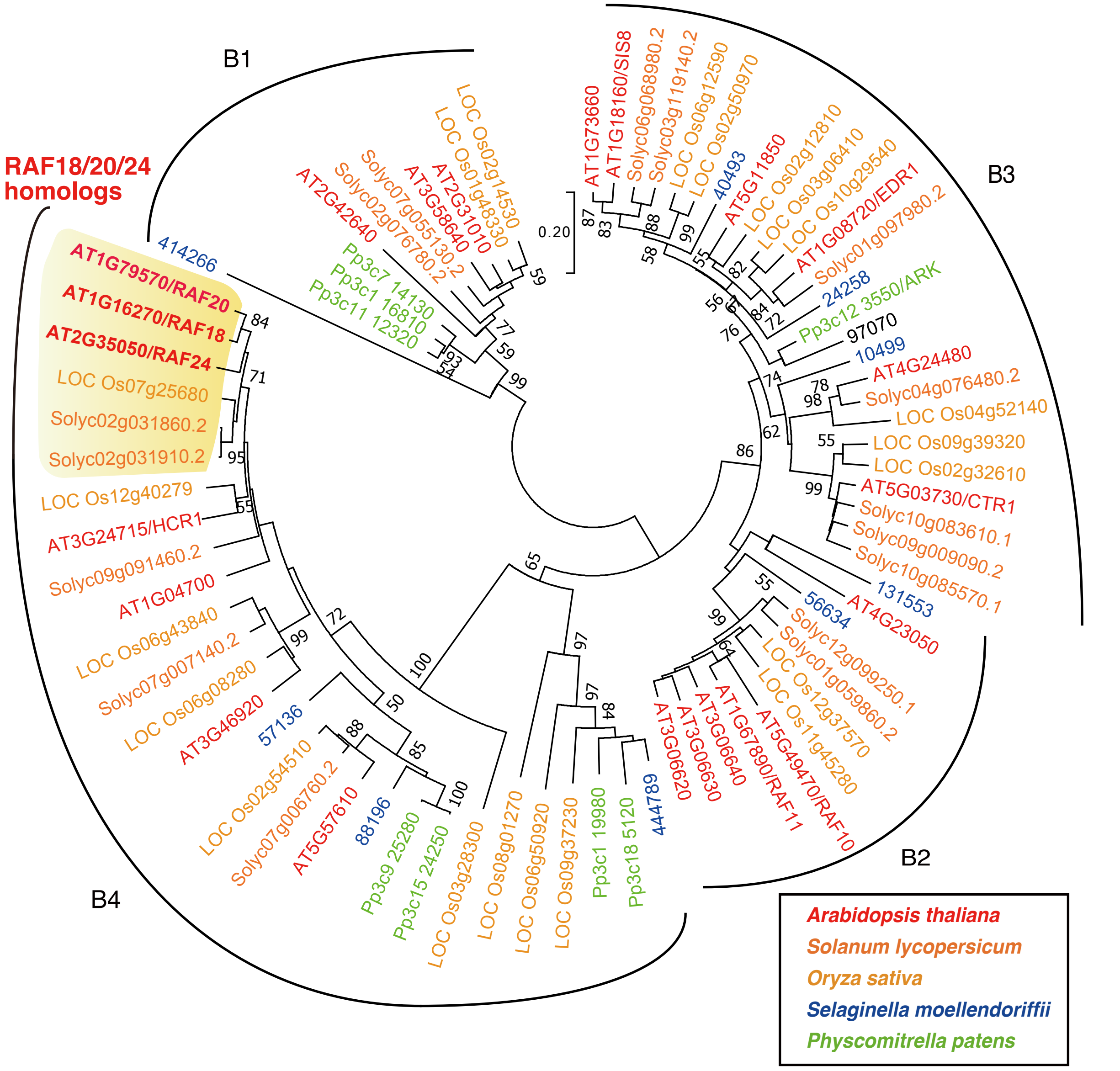

Supplementary Figure 3. Phylogenetic analysis of B4-MAPKKK members from *Arabidopsis thaliana*, *Solanum lycopersicum*, *Oryza sativa*, *Selaginella moellendorffii* (a lycophyte) and *Physcomitrella patents* (a moss).

A neighbour-joining tree was constructed based on alignments of the full-length peptide sequences. The numbers on the side of each branch indicate bootstrap values (≥ 50%) from 1000 replicates. The scale bar indicates the substitution rate site.

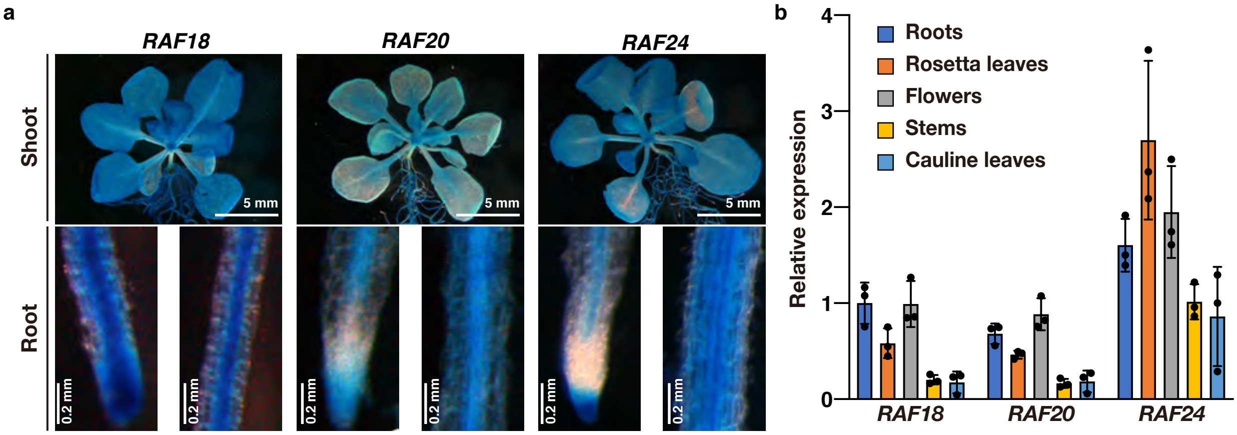

Supplementary Figure 4. Expression analysis of the three *RAF* genes in *Arabidopsis*.

(a) Histochemical localization of GUS activity in transgenic Arabidopsis lines harbouring pGK-RAF18pro-GUS, pGK-RAF20pro-GUS or pGK-RAF24pro-GUS. (b) Expression patterns of Arabidopsis *RAF18, RAF20* and *RAF24* genes in various tissues. The transcript levels of each gene were analysed by quantitative RT-PCR using total RNA extracted from the roots, rosette leaves, flowers, stems and cauline leaves. The expression levels of *RAF18* in the root were defined as 1.0. Values are presented as the means ± s.d. of three biological replicates.

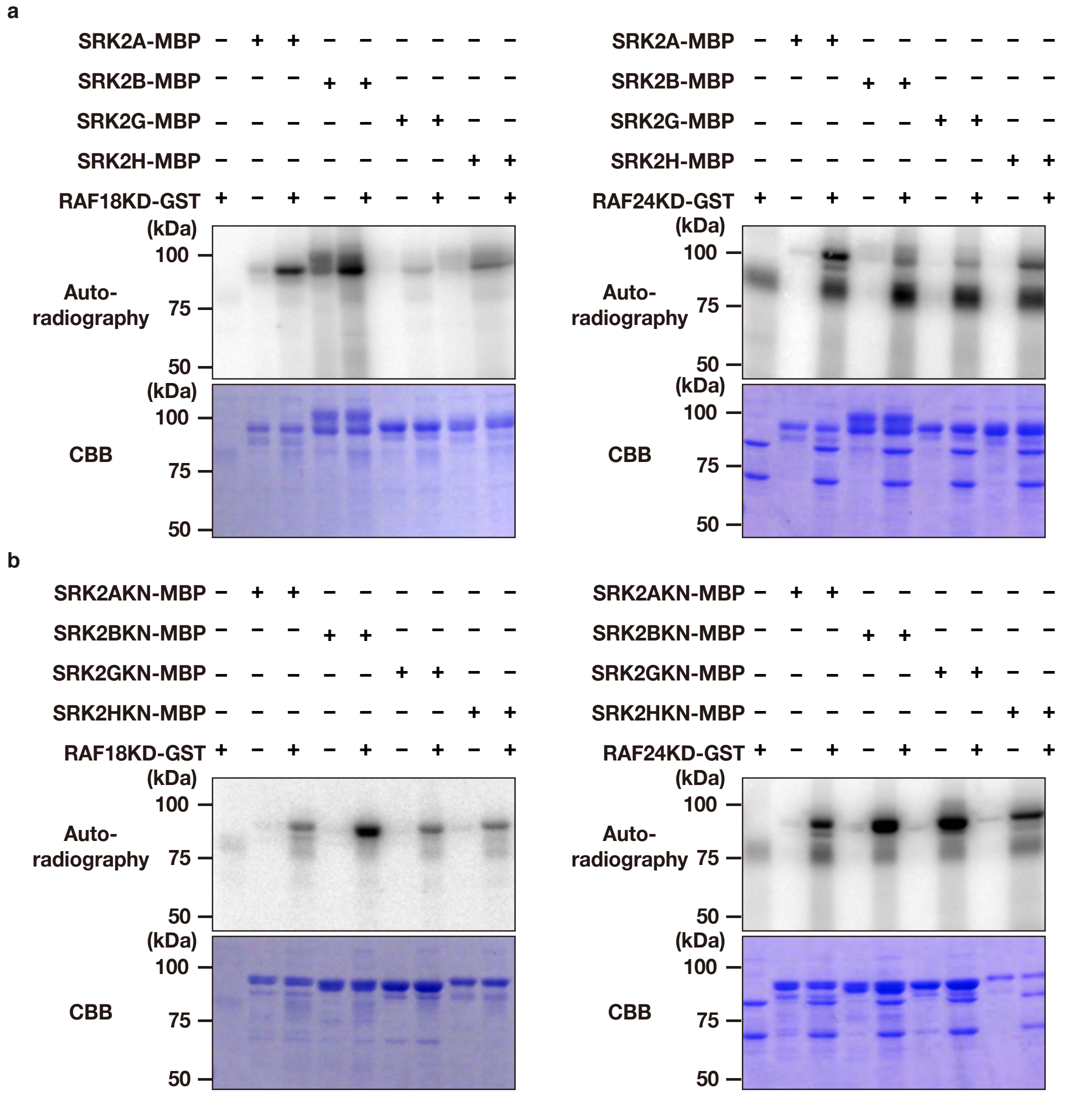

Supplementary Figure 5. RAF18 and RAF24 phosphorylate subclass I SnRK2s *in vitro*.

(a) Phosphorylation of SRK2A-MBP, SRK2B-MBP, SRK2G-MBP and SRK2H-MBP by RAF18KD-GST or RAF24KD-GST *in vitro*. *In vitro* phosphorylation assays were performed using 500 ng of the proteins. Each lane represents an independent reaction in which the indicated combinations of recombinant proteins were tested. Radioactivity-labelled proteins were visualized by autoradiography (Upper panel). Protein abundance was visualized by CBB staining (lower panel). Similar results were obtained in independent experiments. (b) Phosphorylation of SRK2AKN-MBP, SRK2BKN-MBP, SRK2GKN-MBP and SRK2HKN-MBP by RAF18KD-GST or RAF24KD-GST *in vitro*. *In vitro* phosphorylation assays were performed as described in (a).

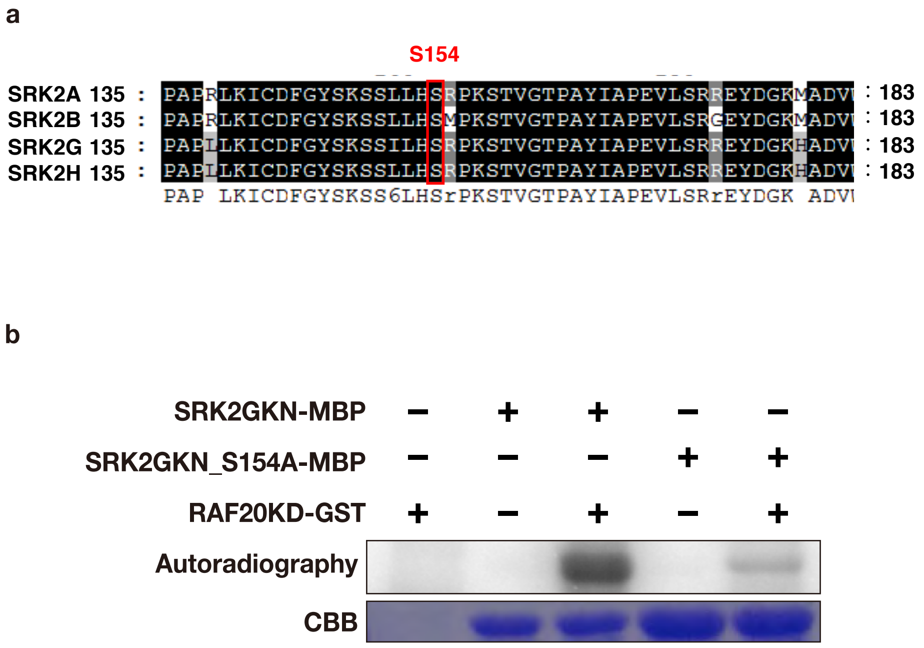

Supplementary figure 6. Identification of phosphorylation sites of SRK2G by RAF20.

(a) An alignment of the amino acid sequences (135-183) in the subclass I SnRK2s. (b) Phosphorylation of SRK2GKN-MBP or SRK2GKN_S154A-MBP by RAF20KD-GST in vitro; 500 ng of the recombinant proteins were reacted with γ-[32P]ATP and electrophoresed on an SDS-polyacrylamide gel, followed by Coomassie Brilliant Blue (CBB) staining and autoradiography.

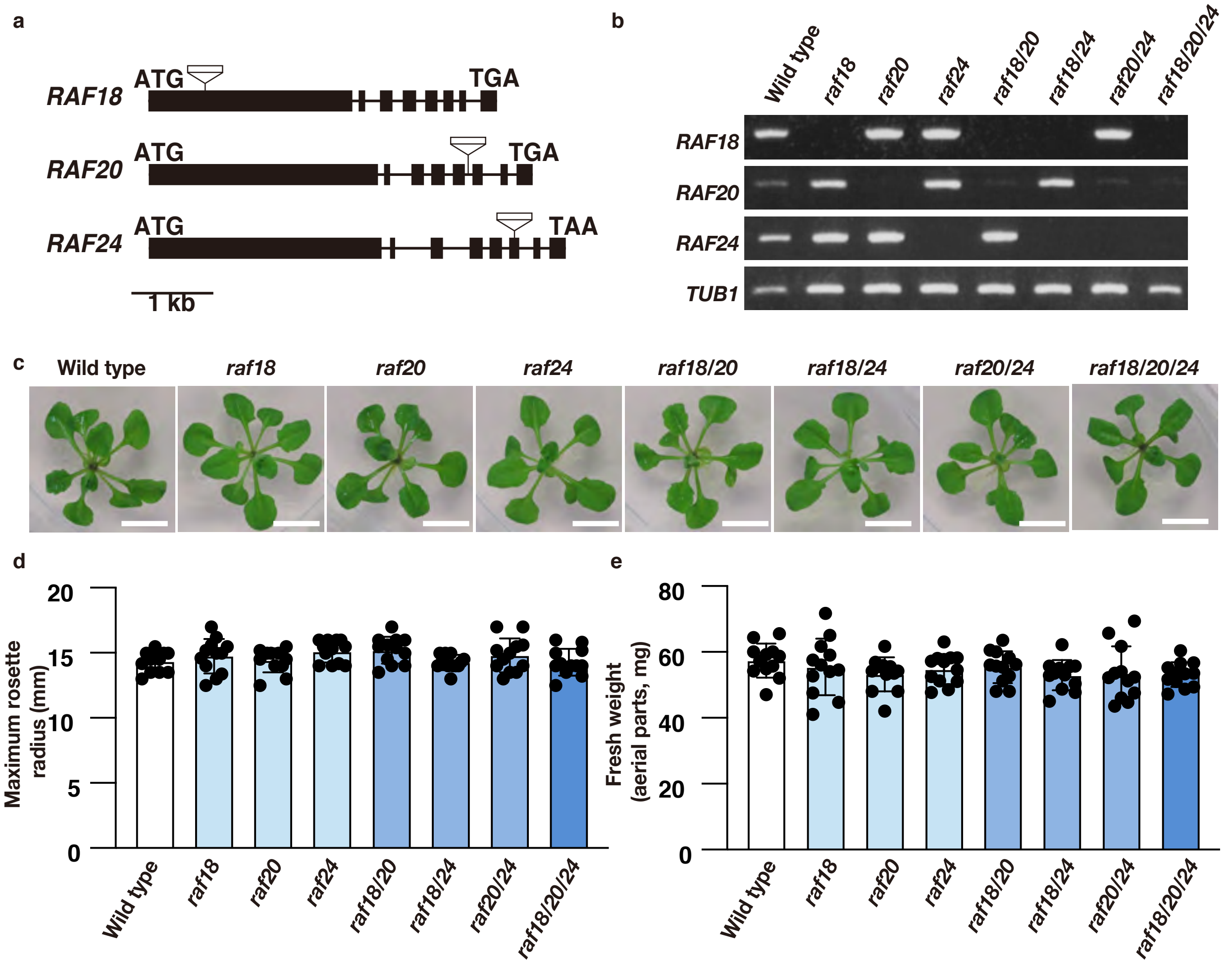

Supplementary Figure 7. Generation of multiple *RAF* mutants and growth phenotypes of multiple *RAF* mutants grown on agar plates.

(a) Scheme of *RAF18*, *RAF20* and *RAF24* genes. Exons, thick lines; introns, thin lines. The positions of the T-DNA insertion in each mutant are shown. (b) The presence or absence of each *RAF* transcript in each *raf* mutant was determined by RT-PCR using *RAF18*-, *RAF20*-, *RAF24*- and *TUB1*-specific primers. (c) Growth phenotypes of plants grown for 3 weeks on agar plates. Scale bars = 1 cm. (d) Maximum rosette radius of each plant grown as described in (c). Bars indicate s.d. (n = 8). (e) Fresh weight of aerial parts of each plant grown as described in (c). Bars indicate s.d. (n = 8).

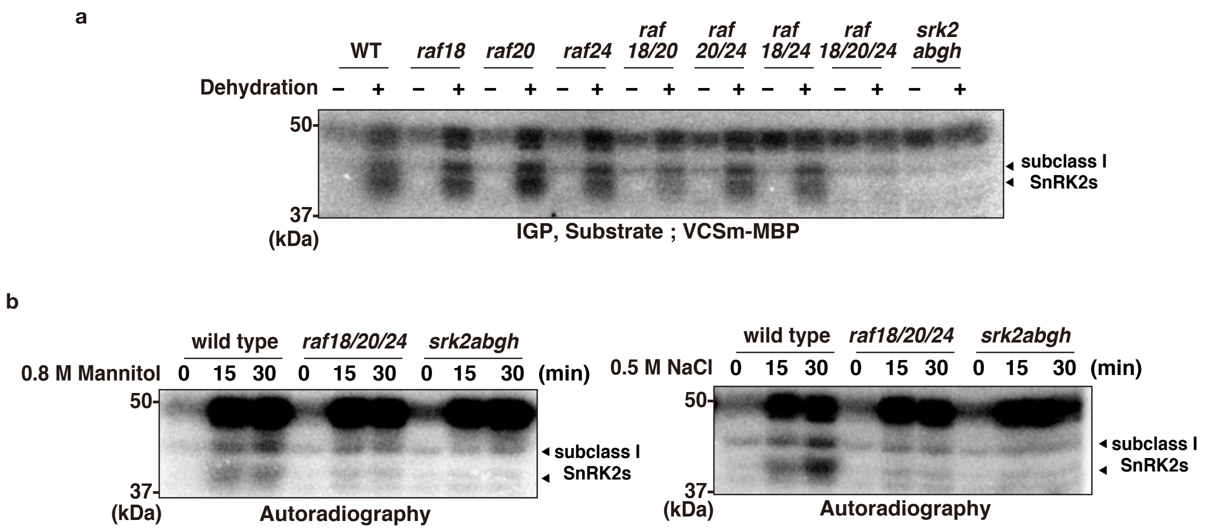

Supplementary Figure 8. Kinase activity of plant native subclass I SnRK2s in *raf* multiple mutants treated with dehydration stress.

(a) Phosphorylation of the VCSm fragment using crude extracts from wild type, *raf* multiple mutant and *srk2abgh* plants treated with dehydration stress, validated using an in-gel kinase assay (IGP). VCSm-MBP was used as a substrate. (b) Phosphorylation of the VCSm fragment using crude extracts from wild type, *raf18/20/24* and *srk2abgh* in response to mannitol or NaCl stress. In-gel kinase assay was performed as described in (a).

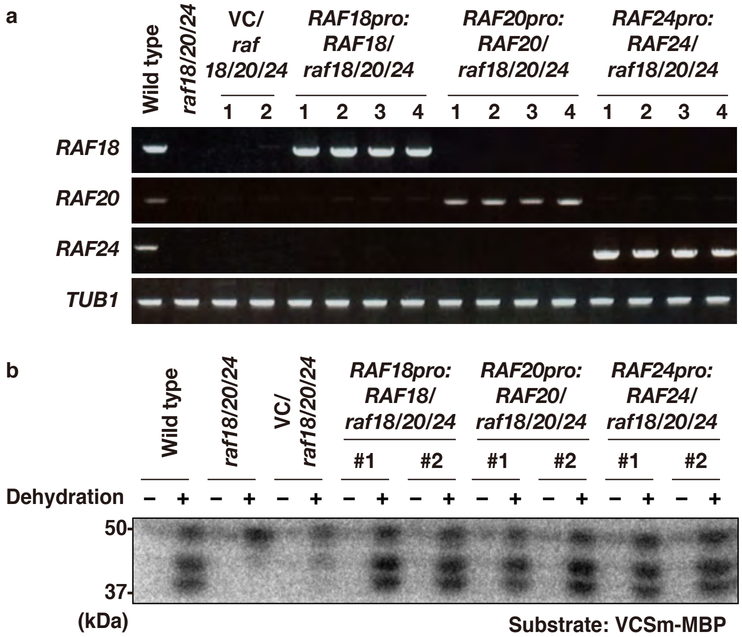

Supplementary Figure 9. Generation of complementation lines of the *raf18/20/24* triple mutant by expressing each *RAF* gene.

(a) *RAF* transcript levels in wild type plants, *raf18/20/24* triple mutants, two independent vector control lines (introducing the vector in the *raf18/20/24* background; VC/*raf18/20/24*), and four independent *raf18/20/24* lines expressing *RAF18*, *RAF20* or *RAF24* each using its own promotor (complementation lines; *RAF18*/*raf18/20/24*, *RAF20*/*raf18/20/24* and *RAF24*/*raf18/20/24*). *RAF* transcripts were detected by RT-PCR using total RNA extracted from 12-d-old seedlings. *TUB1* transcripts were detected as a control in each experiment. (b) Phosphorylation of the VCSm fragment using crude extracts from the wild type, *raf18/20/24*, two independent complementation lines and *srk2abgh* in response to dehydration stress, validated using an in-gel kinase assay (IGP). VCSm-MBP was used as a substrate.

Supplementary Figure 10

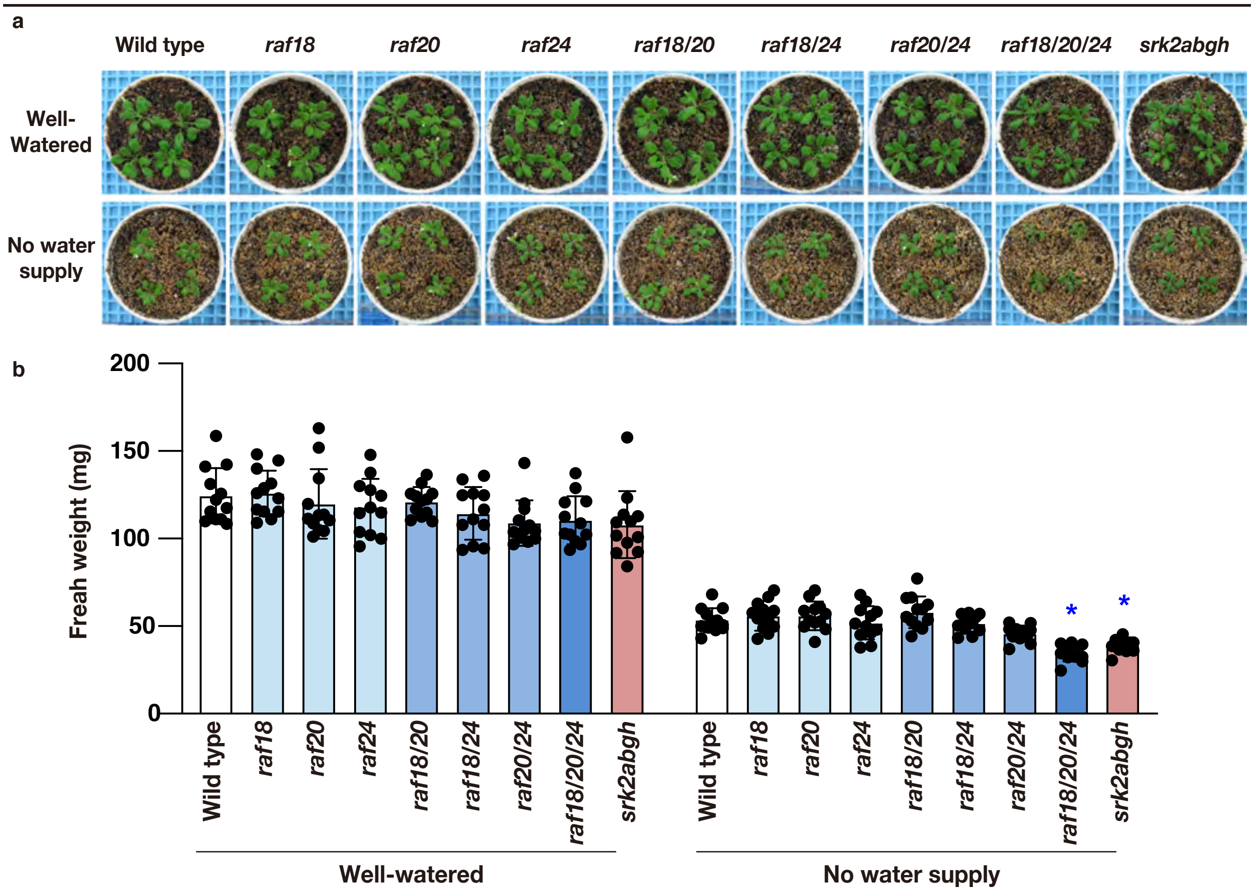

Supplementary Figure 10. Growth phenotypes of *raf* single and multiple mutants under water-deficit stress conditions.

(a) Growth phenotypes of wild type, *raf*-single and multiple mutants, and *srk2abgh* plants grown under water-limited conditions. The plants were grown on germination medium (GM) agar plates for 12 d, in soil for an additional 2 d and subsequently without water for 10 d. (b) Fresh weight (mg) of the aerial parts of plants grown as described in (a). Data represent means ± s.d., n = 12. An asterisk indicates that the corresponding mean is significantly different from the mean value of the wild type within each condition (**P* < 0.05, Dunnett’ s multiple comparison test).

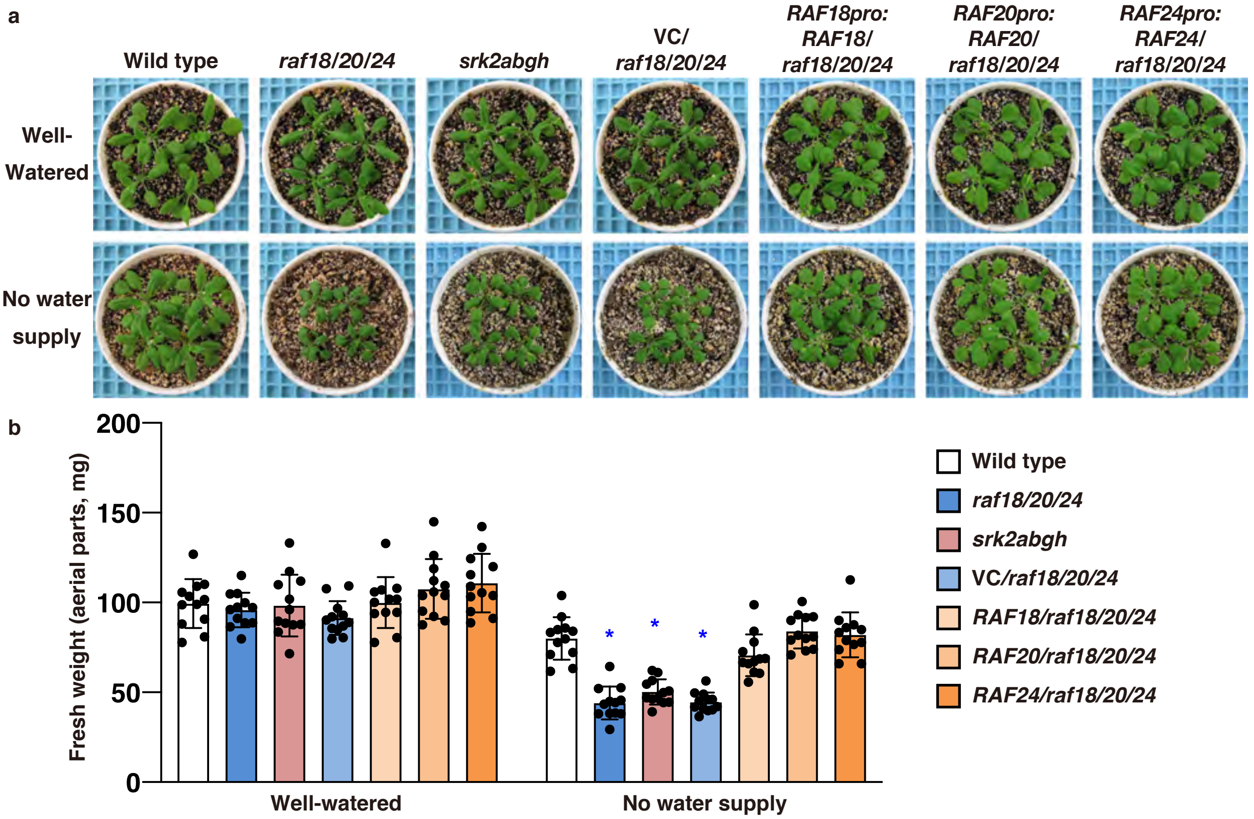

Supplementary Figure 11. Growth recovery of complementation lines of *raf18/20/24* under water-deficit stress conditions.

(a) Growth phenotypes of the wild type, *raf18/20/24*, *srk2abgh*, VC/*raf18/20/24*, *RAF18*/*raf18/20/24*, *RAF20*/*raf18/20/24* and *RAF24*/*raf18/20/24* under water-deficit conditions. (b) Fresh weight (mg) of the aerial parts of plants grown as described in (a). Data represent means ± s.d., n = 12. An asterisk indicates that the corresponding mean is significantly different from the mean value of the wild type within each condition (*$P < 0.05$, Dunnett' s multiple comparison test).

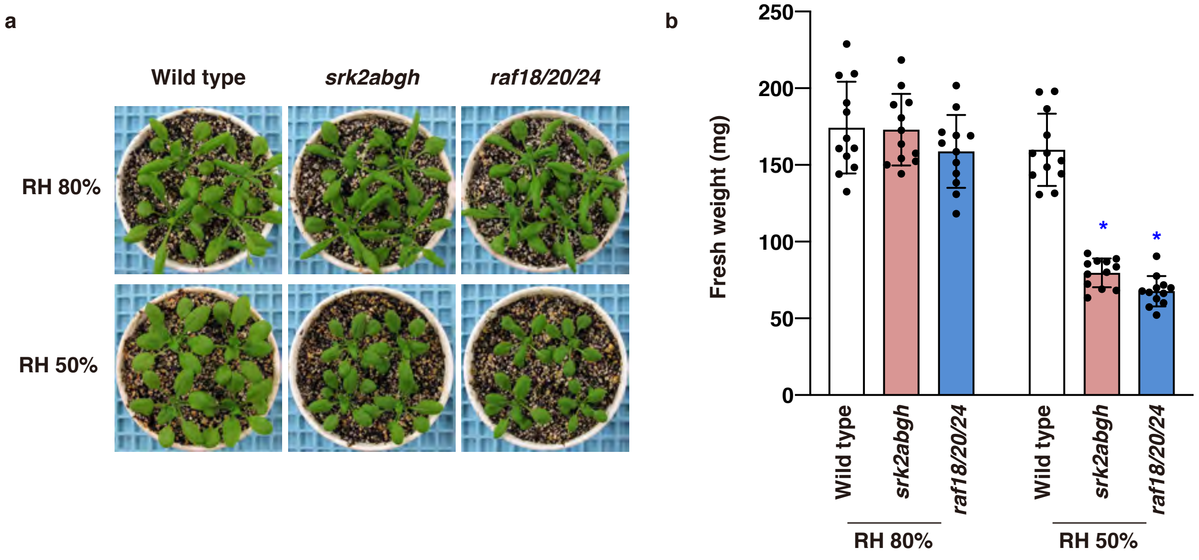

Supplementary Figure 12. Growth of *raf18/20/24* under low-humidity conditions.

(a) Growth phenotypes of the wild type, *raf18/20/24* and *srk2abgh* under low-humidity conditions. Scale bars, 1 cm. (b) Fresh weight (mg) of the aerial parts of plants grown as described in (a). Data represent means ± s.d., n = 12. An asterisk indicates that the corresponding mean was significantly different from the mean value of the wild type within each condition (*$P < 0.01$, two-tailed t-test with Bonferroni correction).

Supplementary Figure 13

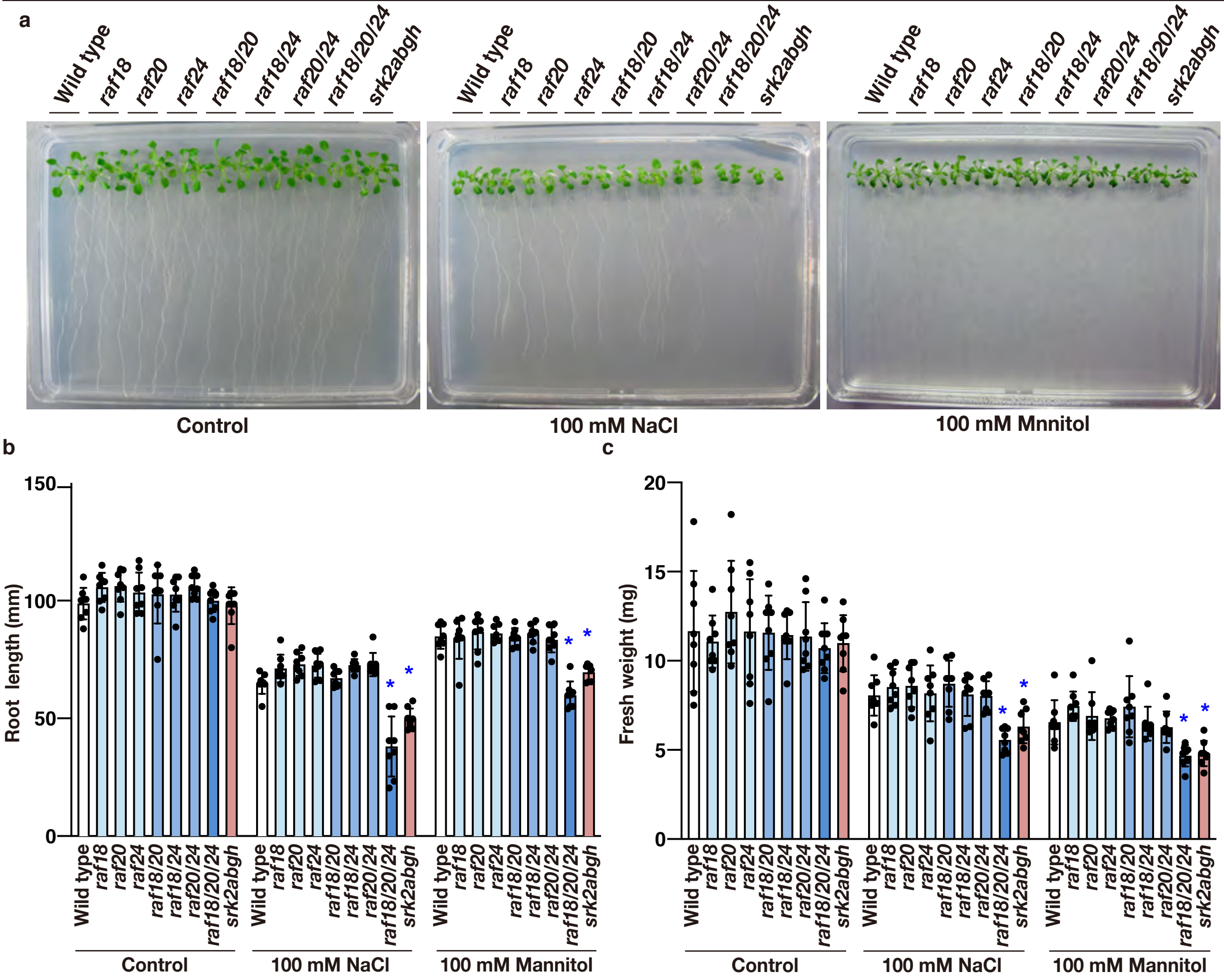

Supplementary Figure 13. Growth phenotypes of *raf18/20/24* under high salinity or mannitol treatment.

(a) Growth phenotypes of the wild type, *raf*-single and multiple mutants, and *srk2abgh* grown on agar plates containing 100 mM NaCl or 100 mM mannitol. Representative data are shown. (b, c) Primary root length (b) and fresh weight (c) of plants grown as described in (a). Data represent means ± s.d., n = 8. An asterisk indicates that the corresponding mean is significantly different from the mean value of the wild type within each condition (*$P < 0.05$, Dunnett' s multiple comparison test).

Supplementary Figure 14

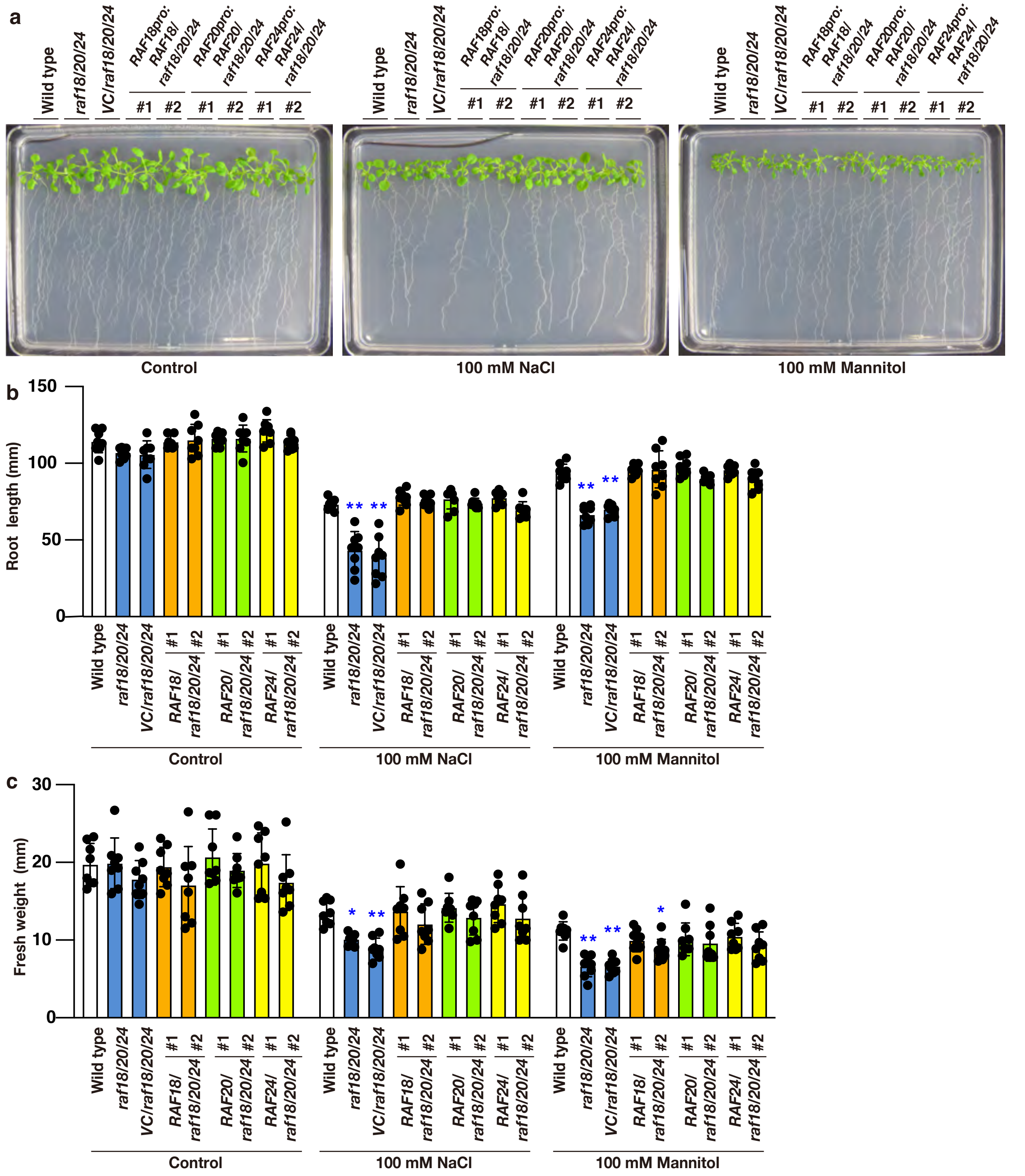

Supplementary Figure 14. Growth phenotypes of complementation lines of *raf18/20/24* under high salinity or mannitol treatment.

(a) Growth phenotypes of the wild type, *raf18/20/24*, VC/*raf18/20/24*, and two independent lines of *RAF18*/*raf18/20/24*, *RAF20*/*raf18/20/24* and *RAF24*/*raf18/20/24* grown on agar plates containing 100 mM NaCl or 100 mM mannitol. (b, c) Primary root length (b) and fresh weight (c) of plants grown as described in (a). Data represent means ± s.d., n = 8. An asterisk indicates that the corresponding mean is significantly different from the mean value of the wild type within each condition (*$P < 0.05$, **$P < 0.01$, Dunnett's multiple comparison test).

Supplemental methods

Histochemical GUS staining

The 1776-bp *RAF18*, 3355-bp *RAF20* and 1419-bp *RAF24* promoter fragments upstream of the translational start site were amplified from Col-0 plants and were then cloned into the pGK-GUS vector[1] to produce the pGK-RAF18pro:GUS, pGK-RAF20pro:GUS and pGK-RAF24pro:GUS constructs, respectively. Transgenic Arabidopsis plants harbouring the pGK-RAF18pro:GUS, pGK-RAF20pro:GUS or pGK-RAF24pro:GUS constructs were subjected to histochemical GUS assays[2]. The primers used for generating the constructs are listed in Supplementary Data 4.

Phylogenetic analysis

The sequences of the B group of Raf-like kinase homologs from *Arabidopsis thaliana*, *Solanum lycopersicum* (tomato), *Oryza sativa* (rice), *Selaginella moellendorffii* (a lycophyte), and *Physcomitrella patens* (a moss) were obtained from the Phytozome database (version 12.1.6; https://phytozome.jgi.doe.gov/pz/portal.html). The peptide sequences were aligned and clustered using Clustal X2.1[3]. The phylogeny of the full-length amino acid sequences was constructed using MEGA7[4]. The distances between branches were calculated using the neighbour-joining method based on 1000 bootstrap samples.

Identification of phosphorylation sites using mass spectrometry

The mass spectrometric analyses were performed using a TripleTOF 5600 instrument (SCIEX) with an Autosampler-2 1D plus and NanoLC Ultra (Eksigent). Each sample was isolated using a MonoCap C18 High-Resolution 2000 column (2000 mm × 1000-μm inside diameter, 2-μm pore size; GL Science, Japan). Eight to ten microliters of the sample was concentrated through the analytical column at a flow rate of 500 nL/min for 30 min. The mobile phase comprised of 2% acetonitrile and 0.1% formic acid (A) and 80% acetonitrile and 0.1% formic acid (B). The following linear gradient was used in the analysis: A:B = 98:2 at 0 min to A:B = 60:40 over 300 min, A:B = 10:90 over 20 min, and A:B = 98:2 over 40 min. The MS scan range was a mass/charge ratio (*m*/*z*) of 400 to 1250, and the top 20 precursor ions were selected for subsequent MS/MS scans in the high-sensitivity mode. The MS/MS data were analysed using ProteinPilot 5.0 software (SCIEX) and subsequently annotated using the *A. thaliana* TAIR 10 protein database for peptide identification.

References

1 Qin, F. *et al.* Arabidopsis DREB2A-interacting proteins function as RING E3 ligases and negatively regulate plant drought stress-responsive gene expression. *Plant Cell* **20**, 1693-1707 doi:10.1105/tpc.107.057380 (2008).

2 Fujita, Y. *et al.* Three SnRK2 protein kinases are the main positive regulators of abscisic acid signaling in response to water stress in Arabidopsis. *Plant Cell Physiol.* **50**, 2123-2132 doi:10.1093/pcp/pcp147 (2009).

3 Larkin, M. A. *et al.* Clustal W and Clustal X version 2.0. *Bioinformatics* **23**, 2947-2948 doi:10.1093/bioinformatics/btm404 (2007).

4 Kumar, S. *et al.* MEGA7: Molecular Evolutionary Genetics Analysis Version 7.0 for Bigger Datasets. *Mol. Biol. Evol.* **33**, 1870-1874 doi:10.1093/molbev/msw054 (2016).
