# Supplementary material for: Plant Raf-like kinases regulate the mRNA population upstream of ABA-unresponsive SnRK2 kinases under drought stress
Source: Nat Commun. 2020 Mar 13;11:1373. doi: 10.1038/s41467-020-15239-3 (PMC7069986; doi:10.1038/s41467-020-15239-3)
Supplement: Supplementary file 3 — Description of Additional Supplementary Files [file 41467_2020_15239_MOESM3_ESM.pdf]

Description of Additional Supplementary Files

Supplementary Data 1. List of candidate subclass I SnRK2-interacting proteins detected in immunoprecipitates from SRK2A-GFP- and/or SRK2G-expressing plants under mannitol treatment.

Criteria

I. The proteins should be identified with a confidence > 95% (ProteinPilot-“unused” >1.3)

II. The proteins should be specifically detected in both the immunoprecipitate from the SRK2A-GFP-expression plants and that from the SRK2G-GFP-expressing plants but not in that from the GFP-expressing plants.

III. The proteins should be detected in two biological samples.

Supplementary Data 2. List of phosphorylated peptides of SRK2G detected by LC-MS/MS analysis.

Supplementary Data 3. List of up- or downregulated genes in the *raf18/20/24* mutant plants subjected to dehydration for 5 h.

Supplementary Data 4. Primer pairs used in this study.
