# Supplementary material for: Plant Raf-like kinases regulate the mRNA population upstream of ABA-unresponsive SnRK2 kinases under drought stress
Source: Nat Commun. 2020 Mar 13;11:1373. doi: 10.1038/s41467-020-15239-3 (PMC7069986; doi:10.1038/s41467-020-15239-3)
Supplement: Supplementary file 8 — Reporting Summary [file 41467_2020_15239_MOESM8_ESM.pdf]

naturereresearch

Corresponding author(s): Kazuko Yamaguchi-Shinozaki

Last updated by author(s): Feb 6, 2020

Reporting Summary

Nature Research wishes to improve the reproducibility of the work that we publish. This form provides structure for consistency and transparency in reporting. For further information on Nature Research policies, seeAuthors & Referees and theEditorial Policy Checklist .

Statistics

For all statistical analyses, confirm that the following items are present in the figure legend, table legend, main text, or Methods section.

n/a	Confirmed	
☐	☒	The exact sample size (*n*) for each experimental group/condition, given as a discrete number and unit of measurement
☐	☒	A statement on whether measurements were taken from distinct samples or whether the same sample was measured repeatedly
☐	☒	The statistical test(s) used AND whether they are one- or two-sided<br>*Only common tests should be described solely by name; describe more complex techniques in the Methods section.*
☒	☐	A description of all covariates tested
☐	☒	A description of any assumptions or corrections, such as tests of normality and adjustment for multiple comparisons
☐	☒	A full description of the statistical parameters including central tendency (e.g. means) or other basic estimates (e.g. regression coefficient) AND variation (e.g. standard deviation) or associated estimates of uncertainty (e.g. confidence intervals)
☒	☐	For null hypothesis testing, the test statistic (e.g. *F*, *t*, *r*) with confidence intervals, effect sizes, degrees of freedom and *P* value noted<br>*Give P values as exact values whenever suitable.*
☒	☐	For Bayesian analysis, information on the choice of priors and Markov chain Monte Carlo settings
☒	☐	For hierarchical and complex designs, identification of the appropriate level for tests and full reporting of outcomes
☒	☐	Estimates of effect sizes (e.g. Cohen's *d*, Pearson's *r*), indicating how they were calculated

Our web collection on statistics for biologists contains articles on many of the points above.

Software and code

Policy information about availability of computer code

Data collection	We used Fiji software(https://fiji.sc/) and Protein Pilot 5.0 to analyze the data.
Data analysis	We used GraphPadPrism 8 to analyze the data. bcl2fastq, bowtie (ver 0.12.9) and PostgreSQL were used for RNA-seq analysis.

For manuscripts utilizing custom algorithms or software that are central to the research but not yet described in published literature, software must be made available to editors/reviewers. We strongly encourage code deposition in a community repository (e.g. GitHub). See the Nature Research guidelines for submitting code & software for further information.

Data

Policy information about availability of data

All manuscripts must include a data availability statement. This statement should provide the following information, where applicable:

- Accession codes, unique identifiers, or web links for publicly available datasets
- A list of figures that have associated raw data
- A description of any restrictions on data availability

The authors declare that all data supporting the findings of this study are avaliable within the article and its Supplementary information files or are available from the corresponding author upon request. The RNA-sequencing data were deposited in DNA Data Bank of Japan under a specific accession number (DRA008643). The LC-MS/MS data were deposited in PRIDE under a specific accession number (PXD017371).

Field-specific reporting

Please select the one below that is the best fit for your research. If you are not sure, read the appropriate sections before making your selection.

Life sciences study design

All studies must disclose on these points even when the disclosure is negative.

Sample size	We did not use statistical methods to determine sample size. Sample size was chosen based on previous studies and experience.
Data exclusions	No data were excluded.
Replication	All attempts at replication were successful.
Randomization	All samples were allocated randomly into experimental groups.
Blinding	We did not apply blinding because the process of sample collection and following analyses were carried out by the same person.

Reporting for specific materials, systems and methods

We require information from authors about some types of materials, experimental systems and methods used in many studies. Here, indicate whether each material, system or method listed is relevant to your study. If you are not sure if a list item applies to your research, read the appropriate section before selecting a response.

Materials & experimental systems

n/a	Involved in the study	
☐	☒	Antibodies
☒	☐	Eukaryotic cell lines
☒	☐	Palaeontology
☒	☐	Animals and other organisms
☒	☐	Human research participants
☒	☐	Clinical data

Methods

n/a	Involved in the study	
☒	☐	ChIP-seq
☒	☐	Flow cytometry
☒	☐	MRI-based neuroimaging

Antibodies

Antibodies used	Anti-GFP(11814460001, Roche)<br>Anti-mCherry([1C51]ab 125096, abcam)<br>Anti-RFP mAb-Magnetic Beads(M165-11, MBL)<br>Anti-GFP MicroBeads(130-091-125,Miltenyi Biotec)
Validation	Information of these antibodies are available in the following web sites.<br>Anti-GFP(11814460001, Roche),(https://www.sigmaaldrich.com/catalog/product/roche/11814460001?lang=ja&region=JP)<br>Anti-mCherry([1C51]ab 125096, abcam),(https://www.abcam.co.jp/mcherry-antibody-1c51-ab125096.html)<br>Anti-RFP mAb-Magnetic Beads(M165-11, MBL),(https://www.mblintl.com/products/m165-11)<br>Anti-GFP MicroBeads(130-091-125,Miltenyi Biotec),(https://www.miltenyibiotec.com/US-en/products/macsmolecular/reagents/protein-research/epitope-tagged-protein-isolation-and-detection/umacs-and-multimacs-gfp-isolation-kits.html)
